# Are vocalists prone to temporomandibular disorders?

**DOI:** 10.1111/joor.12849

**Published:** 2019-07-18

**Authors:** Maurits K. A. van Selms, Jetske W. Wiegers, Frank Lobbezoo, Corine M. Visscher

**Affiliations:** ^1^ Department of Orofacial Pain and Dysfunction Academic Centre for Dentistry Amsterdam (ACTA) University of Amsterdam and Vrije Universiteit Amsterdam Amsterdam The Netherlands

**Keywords:** singing, temporomandibular disorders, temporomandibular disorders pain, temporomandibular joint sounds, work‐related musculoskeletal disorders

## Abstract

**Background:**

As vocalists demand high physical strains of the masticatory system, singing is frequently mentioned as a risk factor for temporomandibular disorders (TMDs).

**Objectives:**

This study investigated whether vocalists report a higher prevalence of two types of TMDs (viz., TMD pain and temporomandibular joint sounds) compared with instrumentalists who do not load their masticatory system while performing. In addition, we examined which risk indicators are associated with the presence of these TMDs among musicians.

**Methods:**

A total of 1470 musicians from 50 different music ensembles completed a questionnaire. Of these musicians, 306 were vocalists (mean age ± SD 37.5 ± 17.7 years; 63.9% female) and 209 musicians enrolled the control group (mean age ± SD 42.7 ± 18.0 years; 40.7% female).

**Results:**

The prevalence of self‐reported TMD pain among vocalists was 21.9%, as compared to 12.0% in the control group. 20.0% of the vocalists reported TMJ sounds versus 15.1% of the controls. The multiple regression models indicated that being a vocalist was not a risk indicator for the presence of self‐reported TMD pain nor for self‐reported TMJ sounds. Instead, it appeared that the report of TMD pain among musicians was positively associated with female gender, next to the level of physical workload, depicted as frequency of oral behaviours and the hours of daily practice. Musicians’ report of TMJ sounds was associated with oral behaviours.

**Conclusion:**

This study shows that singing is not associated with the reports of TMD pain and TMJ sounds, after adjusting for potentially confounding variables included in the models.

## 1 INTRODUCTION

Work‐related musculoskeletal disorders (WMSDs) represent the progression of an overuse injury that can occur in any part of the body associated with movement. WMSDs can be found in a wide array of occupations demanding high physical strains of the employees, ranging from health care to the catering industry.^1^, ^2^ Causative mechanisms behind WMSDs include repetitive motion, forceful exertions, and non‐neutral body postures. Other factors that can aggravate such disorders are high psychosocial work demands and perceived stress.^3^, ^4^ In terms of population exposure, it can be expected that many musicians suffer from WMSDs, because regular training loads resulting from daily practice, rehearsal, and performance place great demands on the neuromusculoskeletal systems of the body. It is therefore not surprising that playing a musical instrument that loads the masticatory system is frequently mentioned as a risk factor for temporomandibular disorders (TMDs)^5^, ^6^.

TMDs are a group of musculoskeletal disorders that comprise clinical problems affecting the masticatory muscles, the temporomandibular joints (TMJs) and associated tissues.^7^ With this in mind, it is frequently suggested that singing is a predisposing factor for TMDs as well.^8^, ^9^, ^10^ The basic idea behind this is that vocalists repetitively submit their masticatory system to unnatural positions during singing, aiming to achieve the desirable sound.^9^ In addition, it can be expected that vocalists are more sensitive to, and aware of, signs and symptoms of TMDs due to the close proximity to their ‘instrument’, the vocal box. Surprisingly, the actual evidence that underlines the idea of singings as a predisposing factor for TMD is very limited.^11^ To the best of our knowledge, only Vaiano et al investigated the presence of 13 types of bodily pains, including TMJ pain, in a group of 50 classical choral singers.^12^ Although there was no significant difference in the presence of this pain when compared to a control group consisting of 150 persons from the general population (non‐singers), it is difficult to extrapolate this specific type of pain to TMD pain in general.

As a part of a large study among musicians in The Netherlands, the aims of the present study were (a) to investigate whether vocalists experience more TMDs (viz., pain‐related forms of TMDs and TMJ sounds) as compared to musicians for whom loading of the masticatory system is not required for the musical performance (eg cellist, percussionist, pianist), and (b) to assess which risk indicators are associated with the presence of these TMDs among musicians.

## 2 METHODS

### 2.1 Data collection

This study was conducted among adult (>18 years) musicians of several music ensembles (symphony orchestras, chamber music ensembles, brass bands, fanfares, and choirs) of different levels of professionalism (from amateur to professional) throughout The Netherlands. Music ensembles were contacted by e‐mail or telephone and were asked whether they would like to participate. Between December 2013 and June 2016, a total of 90 music ensembles (including 15 choirs) were approached to participate in this study. After permission was granted by the chairman, human relations manager, or the conductor of the ensemble to perform the study, each musician that was present received an information letter during one of the rehearsals with details about the survey and a questionnaire. An additional verbal explanation about the project and questionnaire was given to the musicians as well. Participants were asked for written informed consent before completing the questionnaire. This study was considered by the Medical Ethics Review Committee (METc) of the Vrije Universiteit (VU) Medical Centre not to fall under the provisions of the Medical Research Involving Human Subjects Act, and medical ethical approval was granted (reference number 2014.074).

### 2.2 Questionnaire

In order to screen for musculoskeletal complaints in the masticatory system, the ‘Symptom Questionnaire’ (SQ) of the Diagnostic Criteria for Temporomandibular Disorders (DC/TMD)^13^ was implemented in the study questionnaire. The SQ solicits information for the most common types of TMDs (viz., TMD pain and TMJ sounds). Other questions focused on demographics (age, sex), (adverse) oral behaviours (eg grinding, clenching, nail biting)^14^ and psychological aspects (viz., daily stress and feeling depressed or down).^15^, ^16^ In addition, specific musician‐related questions were formulated based on another study on this topic.^17^ These questions aimed to determine the type and number of instruments (including singing) that were played, length of playing experience (years), hours of daily practice and the professional level of the musician. Draft versions of the questionnaire were discussed with colleagues and several musicians to assess whether the questions were unambiguous, and if they provided a good insight into the musculoskeletal loading related to the musical performance. Suggestions for improvement were integrated in the final version of the questionnaire (Table 1).

**Table 1:** Questions included in the questionnaire that was distributed among Dutch music ensembles

Which instrument(s) do you play? [vocalists note ‘singing’] Main instrument: Other instruments:
How long have you been playing your main instrument? (in years)
How much have you played daily, on average, during the last 30 days? (in hours)
What type of musician are you? (amateur or (semi)‐professional)
In the last 30 days, have you had pain in your jaw, temple, in the ear, or in front of the ear on either side? (no, yes)
In the last 30 days, have you had any jaw joint noise(s) when you moved or used your jaw? (no, yes)
What was the overall amount of stress that you experienced during the last 30 days? (NRS 0‐10)
Have you been consistently depressed or down, most of the day, nearly every day, for the last 30 days? (no, yes)
How often did you do the following activities, based on the last 30 days? (5‐point Likert scale, ranging from 0 (‘never’) to 4 (‘always’)) a. grinding during the night; b. grinding during the day; c. clenching during the night; d. clenching during the day; e. nail biting; f. pen biting; and g. gum chewing (average of these seven activities, score between 0 and 4)

### 2.3 Data analysis

From the total sample, two groups of musicians were selected: vocalists, and musicians for whom loading of the masticatory system is not required (eg cellist, percussionist, pianist; regarded as controls). Data from instrumental musicians for whose work loading of their masticatory system while playing their instrument is mandatory (eg violin, oboe, trumpet) were not included in this study. For both vocalists and controls, descriptive statistics were used to summarise the study group characteristics. In order to investigate associations between the group characteristics and the prevalence of self‐reported TMD pain and the prevalence of self‐reported TMJ sounds, logistic regression analyses were used. For both self‐reported TMD pain and self‐reported TMJ sounds (outcome variables), the associations with type of musician (viz., vocalists versus controls) and the other independent variables (viz., gender, age, length of playing experience, hours of daily practice, number of musical instruments, amount of daily stress, feeling depressed or down and frequency of oral behaviours) were evaluated using single regression analyses. All independent variables that showed at least a weak association with the outcome variable (*P*‐value < .10) were incorporated into a multiple regression model to estimate the mutually adjusted effects of predictors on the outcome variable. Predictors with the weakest association with the outcome variable were removed using a backward stepwise approach until all predictors in the final model showed a *P*‐value < .05. The analyses were conducted using the IBM SPSS Statistics 25 software package (IBM Corp, Armonk, NY, USA).

## 3 RESULTS

Of the 1910 eligible musicians, 1470 completed the questionnaire (response rate 77.0%). Of these, 306 musicians were vocalists, and 209 were categorised as controls. In comparison with the control group, the vocalist group comprised more women, and the mean age was lower (see Table 2 for details). The prevalence of self‐reported TMD pain among vocalists was 21.9%, as compared to 12.0% in the control group. TMJ sounds were reported by 20.0% of the vocalists and by 15.1% of the controls.

**Table 2:** Characteristics of the two groups of musicians (mean ± SD for interval and ratio level variables, and percentage for nominal level variables)

	Vocalists (n = 306)	Controls (n = 209)
Age (y)	37.5 ± 17.7	42.7 ± 18.0
Female gender (N, %)	195 (63.9%)	85 (40.7%)
TMD pain (N, %)	67 (21.9%)	25 (12.0%)
TMJ sounds (N, %)	60 (20.0%)	31 (15.1%)
(Semi) Professional (N, %)	133 (43.5%)	73 (35.4%)
Playing experience (y)	18.5 ± 13.9	24.0 ± 15.2
Daily practice (h)	2.1 ± 2.0	2.0 ± 2.0
Playing multiple instruments (N, %)	111 (36.3%)	84 (40.2%)
Amount of daily stress (0‐10)	4.6 ± 2.6	3.6 ± 3.0
Feeling depressed or down (N, %)	34 (11.2%)	10 (4.8%)
Frequency of oral behaviours (0‐4)	0.5 ± 0.5	0.4 ± 0.0.5

The single logistic regression analyses showed a positive association (*P* < .1) between being a singer and reporting of TMD pain. In addition, female gender, having a younger age, being a (semi‐)professional musician, a lower number of years of playing experience, a higher number of hours per day devoted to practise, a higher level of daily stress and a higher frequency of oral behaviours were potentially associated with a report of TMD pain as well (Table 3). In the final multiple regression model, being a singer was not retained. Instead, female gender (OR 2.24, 95% CI: 1.21‐4.13), hours of daily practice (OR 1.16, 95% CI: 1.02‐1.33) and frequency of oral behaviours (OR 3.04, 95% CI: 1.86‐4.97) were best associated with the report of TMD pain.

**Table 3:** Single and multiple logistic regression models of variables associated with TMD pain among musicians (n = 515)

Independent variable	Single regression models		P‐to‐Exit	Multiple regression model	
	*P* value	OR (95% CI)		*P* value	OR (95% CI)
Type of musician					
Control group		1			
Vocalists	.004	2.06 (1.25‐3.40)	.115	–	–
Gender					
Male		1		1	
Female	.013	1.82 (1.14‐2.93)		.010	2.24 (1.21‐4.13)
Age (y)	<.001	0.97 (0.96‐0.99)	.072	–	–
Professionalism					
Amateur		1			
(Semi)professional	.011	1.81 (1.15‐2.85)	.835	–	–
Playing experience (y)	.068	0.99 (0.97‐1.00)	.135	–	–
Daily practice (h)	.025	1.13 (1.02‐1.25)		.026	1.16 (1.02‐1.33)
Playing multiple instruments					
No		1			
Yes	.782	1.07 (0.67‐1.70)			
Stress (0‐10)	.004	1.13 (1.04‐1.23)	.362	–	–
Feeling depressed or down					
No		1			
Yes	.193	1.62 (0.78‐3.33)			
Oral behaviours (0‐4)	<.001	3.05 (1.91‐4.88)		<.001	3.04 (1.86‐4.97)

Note

Associations are expressed as odds ratios (OR) and 95% confidence intervals (CI). For each removed independent variable, the *P*‐to‐Exit is reported.

In Table 4, the outcomes of the single and multiple logistic regression analyses with respect to the report of TMJ sounds are presented. It appeared that female gender, having a younger age and a higher frequency of oral behaviours were positively associated with TMJ sounds in the single regression models, of which only the variable ‘oral behaviours’ (OR 2.24, 95% CI: 1.40‐3.57) was retained in the final model.

**Table 4:** Single and multiple logistic regression models of variables associated with TMJ sounds among musicians (n = 515)

Independent variable	Single regression models		P‐to‐Exit	Multiple regression model	
	*P* value	OR (95% CI)		*P* value	OR (95% CI)
Type of musician					
Control group		1		
Vocalists	.163	1.40 (0.87‐2.26)			
Gender					
Male		1			
Female	.077	1.52 (0.96‐2.43)	.176	–	–
Age (y)	.005	0.98 (0.97‐0.99)	.205	–	–
Professionalism					
Amateur		1			
(Semi)professional	.157	1.39 (0.88‐2.20)			
Playing experience (y)	.433	0.99 (0.98‐1.01)			
Daily practice (h)	.448	1.05 (0.93‐1.17)			
Playing multiple instruments					
No		1			
Yes	.418	1.21 (0.76‐1.92)			
Stress (0‐10)	.100	1.07 (0.99‐1.17)			
Feeling depressed or down					
No		1			
Yes	.352	1.43 (0.68‐3.01)			
Oral behaviours (0‐4)	.001	2.24 (1.40‐3.57)		.001	2.24 (1.40‐3.57)

Note

Associations are expressed as odds ratios (OR) and 95% confidence intervals (CI). For each removed independent variable, the P‐to‐Exit is reported.

## 4 DISCUSSION

Temporomandibular disorders (TMDs) is a broad term, used to characterise pain and functional complaints originating from the masticatory system (ie the TMJ, masticatory muscles, or both).^18^ The complaints usually fluctuate and are function dependent.^7^, ^18^ Although regular use of the masticatory system will not necessarily lead to complaints related to TMDs, it is believed that playing a musical instrument often requires mandibular activities that are beyond normal physiological function.^19^ Besides physiological overloading, many epidemiological studies have demonstrated the existence of an association between TMDs and psychopathology.^20^ As performing musicians face various sources of psychological stress due to the demanding and high competitive work demands,^21^, ^22^ it might thus be expected that musicians are especially vulnerable to TMDs. However, the authors of several literature reviews concluded that the low methodological quality and large heterogeneity of the available studies prevented drawing firm conclusions whether playing musical instruments really forms a risk for TMDs.^11^, ^23^, ^24^ Furthermore, except for a study that examined bodily pains in classical choral singers, including pain in the TMJ,^12^ it has never been investigated whether singing is a risk indicator for TMDs. Therefore, the present study investigated whether vocalists experience more TMDs (viz., self‐reported TMD pain and self‐reported TMJ sounds) as compared to musicians for whom loading of the masticatory system is not required for the musical performance. In addition, it was investigated whether specific musician‐related factors played a role in their complaints. Initially, the unadjusted results of this study showed a positive association between being a singer and the report of TMD pain; for the report of TMJ sounds, being a vocalist appeared not to be a risk indicator. The initial association between singing and TMD pain lost significance in the multiple regression model after correction for the influence of gender, hours of daily practice and oral behaviours. This means that the observed higher occurrence of these pain complaints among singers (viz., 21.9%) as compared to controls (viz., 12.0%) might be explained by differences between the groups in gender distribution, daily practice and oral behaviour report. Indeed, the vocalist group comprised significantly more women than the control group. As women seem to be more affected with TMD pain than men,^7^, ^25^ it is essential to consider gender as confounding variable in this type of studies.

Interestingly, the present study indicates that musicians reported more TMD pain in case they had practised more hours on a daily basis. This coincides with knowledge on the field of work physiology, namely that the length of daily working hours and perceived physical workload are risk factors for the development of work‐related musculoskeletal disorders (WMSDs).^3^, ^4^ In line with this is the current finding that the factor ‘oral behaviours’ was the strongest predictor for the presence of self‐reported TMD pain and self‐reported TMJ sounds among musicians. This was not surprising, because a commonly held view in the literature and clinical practice is that TMDs may be caused by mechanical overloading. Both heavy forces and less heavy forces (eg prolonged clenching) may lead to overloading of the jaw‐closing muscles and compressive forces within the TMJ, and thus to TMD pain and joint sounds, respectively.^26^, ^27^, ^28^ However, it has to be reminded that support for this association mainly comes from questionnaire studies, and rarely comes from studies using instrumental techniques.^29^ As questionnaire studies only indicate associations and not necessarily the direction of the relationship, it is impossible to establish how reports of oral behaviours and TMD complaints are associated. In fact, it might be hypothesised that patients, who experience complaints in the oro‐facial area, attribute these complaints to certain factors of which they believe are causal, such as stress or oral behaviours. It might also be possible that a person with a popping TMJ sound or a nagging pain in the masticatory muscles is more aware of oral behaviours than someone who is free of such symptoms. In other words, the presence of complaints in the oro‐facial area could actually drive self‐reporting of oral behaviours. Future studies are needed to more fully explore the underlying mechanisms of the relationship between oral behaviours and TMDs.

A drawback of the current study deals with the fact that the selection of the two groups of musicians was based only on the question inquiring for the main instrument. However, it turned out that almost 40% of the musicians played more than one musical instrument (including singing). Even though the variable ‘Playing multiple instruments’ was not associated with the presence of self‐reported TMD pain and self‐reported TMJ sounds among musicians, the influence of this possible selection bias cannot be ruled out. Another limitation of the present study deals with its cross‐sectional nature. As discussed before, the observed findings merely reveal associations that require further testing. Another drawback deals with the subjective nature: both TMD pain and TMJ sounds were assessed through self‐report only. However, the question that screened for TMD pain is implemented in the validated DC/TMS Axis I protocol,^13^ and is very similar to a TMD pain screening question that exhibited excellent content validity.^30^ Since self‐reported TMJ clicking has been found to be associated with objectively recorded TMJ clicking as well,^31^ it was assumed that this self‐report aspect may not have radically influenced the outcome. On the other hand, the self‐report of oral behaviours represents a different challenge. Ideally, oral behaviours like bruxism activities should be measured using instrumental recording techniques.^28^ However, as the studied items represented such a wide range of oral behaviours, recording them all is deemed impossible. Fortunately, concurrent validity of the seven items, derived from the Dutch Oral Parafunctions Questionnaire, with the Oral Behaviours Checklist that is implemented in the DC/TMD,^32^ appeared to be good.^33^

In summary, the present study indicates that singing was not associated with the reports of TMD pain and TMJ sounds, after adjusting for potentially confounding variables included in the models. The best predictors for self‐reported TMD pain among musicians were female gender, next to the level of physical workload, depicted as frequency of oral behaviours and the hours of daily practice. Musicians’ report of TMJ sounds was associated with oral behaviours.

## CONFLICTS OF INTEREST

None declared.
